# Conference Report: 16th NATIONAL COMPUTER SECURITY CONFERENCE Baltimore, MD September 20–23, 1993

**DOI:** 10.6028/jres.099.061

**Published:** 1994

**Authors:** Dennis Gilbert

**Affiliations:** Computer Security Division, National Institute of Standards and Technology, Gaithersburg, MD 20899-0001

## 1. Introduction

Each year the National Institute of Standards and Technology and the National Computer Security Center (NCSC) cosponsor the National Computer Security Conference. The conference provides a forum for technology interchange among system developers and a place where computer users can exchange ideas and learn new ways to apply current computer and information security technology. This major event on the computer security conference calendar provides an excellent opportunity for attendees to hear the leaders in the field report on their research and share experiences. A large, diverse national and international audience attends the conference. This past year, nearly 2000 attended from government, industry, and academe. One of the most important aspects of the conference is that its many activities provide an opportunity for contemporaries to network and gain new perspectives through the sharing of information and experiences.

## 2. Conference Program Highlights

In addition to a track on criteria and evaluation, there were also tracks on research and development, integration and applications, and management and administration. Each track provided 11 sessions, each of 1 1/2 hours, of peer-reviewed papers or panel sessions. The opening and closing plenary sessions presented subjects and issues of interest and importance to the community. An added highlight of the conference was a tutorial track for newcomers to the computer security field.

### 2.1 Criteria and Evaluation Track

A full track devoted to federal criteria, evaluation, and international harmonization efforts was featured at this 16th anuual conference program. Collaborative efforts by NIST and the National Security Agency (NSA) to develop new criteria for trused systems were presented. Thses criteria will be used to evaluate the ability of system to protect confidentiality of data and provide other security controls. The evolutionary efforts of the commission of European Communities (EC) are focused on producing a comprehensive set of security requirements for designing and developing trust technology for widespread international use. The work performed by the United State and the EC to develop a common basis for product evolution will serve to reduce costs to users, and vendors. Through tutorials, progress reports, and forums, the track placed in perspective the relationships among U.S. and European efforts and their short- and long-term significance

### 2.2 Research and Development Track

Papers and panels in this track typically address technical R&D efforts related to security models. As in the past, a major interest in this track was the various aspects of the subject of access control, i.e., the rules, policies, and mechanisms that address which persons or which computer processes have access to a computer’s data and resources, and under what circumstances. Access control-related papers covered: nonrepudiation in open telecooperation; a mandatory denial of service model; issues and research directions in applying discretionary access control in object-oriented databases; new perspective on access control policies; regulating processing sequences through object state to achieve access control; and a proposed model and related security policy for a mechanism used to provide access to Internet protocol networks.

Two other papers addressed referential integrity and query acceleration in multilevel secure database systems. Still another presented an improved method of checking for “bad passwords” (See Sec. 4).

To share the learnings of other forums, Hilary Hosmer, Data Security, Inc., chaired *Best of New Security Paradigms II Workshop Panel*. This panel examined: the relationship of responsibility modeling and security requirements, a paradigm for flexible and adaptable access control in distributed applications, and identification and authentication when users have multiple accounts. The *Enterprise Security Solutions Panel*, chaired by Paul Lambert Motorola, looked at security from an “enterprise” perspective, covering such areas as business issues and information security, securing the world’s largest private internet, security with token-based access controls, and secure distributed computing for heterogeneous operating system environments. Three other panels presented research or concepts concerned with trusted applications. These included *Strategies for Integrating Evaluated Products* chaired by Dr. James G. Williams, The Mitre Corp.; *Multilevel Information System Security Initiative (MISSI)*, chaired by Gary Secrest, NSA; and *Trusted Applications*, chaired by Janet Cugini, NIST.

### 2.3 Integration and Applications Track

This track focuses on how security technology is being applied and how security products are being evaluated and integrated into secure systems. One of the themes in this track was that of certification and accreditation (C&A), i.e., the evaluation of the technical and nontechnical security controls to: determine whether a specified set of security requirements are met; and support an official authorization by an appropriate management approving authority to place a system employing a prescribed set of safeguards into operational use. Papers in this area covered such subjects as C&A in a military communications network and in an Army multilevel secure (MLS) management information systems environment. Two other papers presented an approach and comprehensive methodology to C&A. Also, a panel in this track presented an update on INFOSEC design and certification initiatives at NSA.

In addition to the papers and panel described, another focus area in this track was network security. Panels in this area included *Network Security Management—The Harder Problem*, chaired by Ronda Henning, Harris Corporation, and *Application of INFOSEC Products on Wide-Area-Networks*, chaired by Joyce Capell, Lockheed Missiles & Space Company, Inc.

Still another set of papers explored the subjects of access control policy needs among federal and private sector organizations and the administration of access rights in a multi-vendor system. The use of commercial-off-the-shelf products for automated information systems (AIS) security, choosing a standard for security protocols, and performing product and system evaluations were also featured in this track, as were such topics as distributed auditing, an approach to risk assessment, designing secure MLS database systems, system testing, and integration of trusted products.

A panel presenting a *Debate of Critical Player Perspectives on MLS System Solution Acquisition Topics*, chaired by Joel E. Sachs, Area Systems, Inc., and one on *Security Issues for the Securities Industry*, chaired by Sally Meglathery, New York Stock Exchange provided the benefit of practical experience to track attendees.

### 2.4 Management and Administration Track

This track presented a variety of papers and panel discussions on issues of concern in the management and administration of automated information systems and the security function.

A particularly interesting and thought-provoking paper in this track explored the applicability of social psychology to information security (See Sec. 4).

Two other panels—On *a Better Understanding of Risk Management Techniques*, chaired by Stuart W. Katzke, NIST; and “*How Much Security is Enough?” The Accreditor’s Perspective*, chaired by James P. Litchko, Trusted Information Systems, Inc. presented management considerations regarding risk management and accreditation. A related paper, *Trusted Systems: Applying the Theory in a Commercial Firm*, by Ernest C. Charles, Donna A. Diodati, and Walter J. Mozdzierz, Aetna Life & Casualty, showed an application of trusted systems in a commercial environment.

A particularly lively panel in this track, *Protection of Intellectual Property*, chaired by Gerald S. Lang, Harrison Ave. Corp., explored the technical, privacy, ethical, legal, political, and piracy issues involved in defining and protecting intellectual property. Another set of panels looked at planning for and responding to emergency situations. *Terror at the World Trade Center*, chaired by Sally Meglathery, New York Stock Exchange, discussed the security issues raised by the World Trade Center bombing. *Contingency Planning in the 90s*, chaired by Irene Gilbert, NIST, examined new technologies and innovative approaches to the subject from an organizational, service provider, and user perspective—with emphasis on the planning process and need to focus senior management attention.

Still another series of panels examined a related set of subjects. These included: *The Privacy Impact of Technology in the 90s*, chaired by Wayne Madsen, Computer Sciences Corporation; *Electronic Crime Prevention*, chaired by Robert Lau, National Security Agency; *Virus Attacks and Counterattacks: Real-World Experiences*, chaired by James P. Litchko, Trusted Information Systems, Inc.; and *Security and Auditability of Electronic Vote Tabulation Systems*, chaired by Rebecca Mercuri, University of Pennsylvania.

*Security Awareness, Training, and Professionalization: Status Report*, chaired by Dennis Gilbert, NIST presented a report by representatives of Key organizations with a stake in promoting security training and professional development in the Federal government and the private sector, A related paper that addressed how to raise awareness about security issues was *How to Market the Information Systems Security Program*, by David Eakin, CISSP, Naval Ships Parts Control Center.

Another interesting panel, *The OECD guidlines for the Security of Information Systems: A Look to the Future*, chaired by Christine Axsmith, Esq., ManTech Strategic Associates, Ltd., reported on the efforts of the Organization for Economic Cooperation and Development to establish a common international framework for computer security

The goal of the guidelines is to develop a common set of principals from which many nations can begin to develop their computer security awareness and practices. It is expected that the guidelines will foster the proliferation of international trade.

### 2.5 Tutorials and Presentations Track

A feature of each conference is a tutorial track. It provides those new to the field, those new to a particular subject, and those who are experienced practitioners wanting a “refresh,” an opportunity to get a basic review of a given security subject. This year’s conference provided a tutorial series on trusted systems, which covered a threats and security overview, trusted systems concepts, trusted networks, trusted database systems, and trusted integration. The tutorial portion of the track also included a session on viruses. In addition, the track offered a panel session on *Getting Your Work Published*, chaired hy Jack Molleran, National Security Agency, and another on *Information Systems Security Standards, The DISA Process*, chaired by Bill Smith, CISSP, DISA. The former presented insights and “tips” hy successful authors of security-related publications. The latter described the role of the Defense Information Systems Agency in the DoD INFOSEC standardization process. A final panel in this track, *Security Requirements for Cryptographic Modules*, chaired by Lisa Carnahan, NIST, gave information on the applicable NIST standards and validation program.

### 2.6 Closing Plenary

Of particular interest was the closing plenary session in which Thomas R. Malarkcy. Department of Defence, presented a paper entitled *Seven Strategies for Information Techinology Protection in the 1990s*, which attempted to lay out several useful strategies for improving the U.S. posture in information technology (IT) security. The first section of the paper presented the author’s view of the success and failures of IT security in the 1980s. The second section identified IT trends and the IT infrastructure that needs to he protected over the next decade. The third section described seven suggested directions for government and industry. These include: 1) establishing a national IT protection policy; 2) promulgating a national policy on minimum IT protection requirements; 3) unifying the current security and safety communities into one protection community; 4) improved emphasis on system security management; 5) examining alternatives to current traditional in-depth evaluation for product quality control; 6) improving accountability features of IT products; and 7) increasing our investment in security interoperability. The presentation of the paper was followed by a lively discussion by the previous recipients of the conference award for outstanding contributions to the field. (See Sec. 3). Chairing the session was award recipient, Stephen Walker, and fellow recipients, James P. Anderson, Dr. Roger Schell, and Dr. Willis Ware.

## 3. Outstanding Contributions to the Field

Each year the conference presents an award to an individual who has made significant contributions to the computer security community over a period of years. This year’s recipient was Mr. Robert C. Courtney, of RCI, Inc. He was IBM’s first Director of Data Security, Privacy, and Integrity. At IBM he launched a number of far-reaching research and development programs. Since 1981 he has been an independent security consultant and has testified frequently before Congress on data security-related matters.

## 4. Outstanding Papers

Also presented this year were two outstanding paper awards. One went to Michel E. Kabay, Ph.D., of the National Computer Security Association for his paper “Social Psychology and INFOSEC: Psycho-Social Factors in the Implementation of Information Security Policy.” Significantly, the paper extends to computer security the learnings of another discipline — that of social psychology. By tapping into advice from the so-called “soft sciences,” the paper argues that improving security depends on changing beliefs attitudes, and behaviors of individuals and organizations. It shows how social psychology can help us to best work with human predilections and predispositions to achieve computer security goals. The other outstanding paper award went to Chris Davies and Ravi Ganesan of Bell Atlantic for their submission “BApasswd: A New Proactive Password Checker.” This paper describes a process that can help people choose passwords that are less likely to be vulnerable to dictionary attack. Some people point to poorly chosen passwords as the single largest cause of security incidents. Of significance is that the authors present an approach that minimizes storage and time to examine chosen passwords.

## 5. Awards Ceremony

This year, as in past years, the conference held a joint awards ceremony in which NIST and NCSC honored the vendors who had successfully developed products meeting the standards of their respective organizations. In the case of NIST, its Computer Security Division provides validation services for vendors to use in testing devices for conformance to security standards defined in three Federal Information Processing Standards (FIPS): Data Encryption Standard (DES), Computer Data Authentication, and Key Management Using ANSI X9.17. In the case of NCSC, vendors are recognized who contribute to the availability of trusted products and who thereby expand the range of solutions customers can use to secure their data. The products are placed on the Evaluated Products List (EPL) following a successful evaluation against the Trusted Computer Systems Evaluation Criteria and its interpretations. (For further information, contact 301-975-2920 regarding the NIST awards and 410-859-4371 regarding the NCSC awards.)

## 6. Other Activities of Interest

In addition to the main track sessions, a number of other activities were available to the conference attendees including:

- Booths featuring NIST publications and NSA information security (INFOSEC) awareness activities. The NIST booth highlighted the NIST Computer Systems Laboratory Bulletins, 4 to 12 page documents, each of which covers a security topic in depth. The NSA booth highlighted NSA technical security guidelines, known as the Rainbow Series, named for the variety of its brightly colored document covers.
- A book exhibit representing a selection of leading publishing firms and the latest selections in published books on computer security.
- Demonstrations of the NIST Computer Security Bulletin Board and NSA’s Dockmaster provide a wide variety of computer security information to federal agencies and to the public. Information posted on the NIST BBS includes an events calendar, software, reviews, publications, bibliographies, list of organizations, and other government bulletin board numbers. Also featured is a set of advisories providing up-to-date information on computer security incidents and how to respond to them. Dockmaster is the focal point for nationwide dissemination and exchange of INFOSEC data through electronic mail and BBSs. Over 2000 users from federal government organizations, private companies, and academic institutions participate in its forums and retrieve data on INFOSEC products, conferences, and training.
- An overview of Air Force systems security initiatives and the status of the initiatives covering incident response, online surveys, and trends in tool development, with emphasis on tools to enhance security on systems and in organizations. Also, demonstrations of tools on intrusion detection, risk management, and training.
- “Networking” rooms for informal and “spur of the moment” discussions away from the crowded hallways.
- An evening reception following the vendor awards ceremony and a banquet, at which a distinguished member of the community provided a light-hearted, but thoughtful view of the profession in an after dinner talk. This year’s speaker was Cheryl Helsing of Sun Microsystems, who has had considerable experience in many aspects of information systems security.

## 7. Future Conferences

It is expected that the next several National Computer Security Conferences will be held in the fall of each year in Baltimore, Maryland.

## 8. To Obtain the Conference Proceedings

Single copies of the 542-page NCSC16 conference proceedings are available upon request. Please contact NIST CSL Publications at 301-975-2821.

